# Reply to Chao et al. Comment on “Nahok et al. Monosodium Glutamate Induces Changes in Hepatic and Renal Metabolic Profiles and Gut Microbiome of Wistar Rats. *Nutrients* 2021, *13*, 1865”

**DOI:** 10.3390/nu14204387

**Published:** 2022-10-19

**Authors:** Kanokwan Nahok, Carlo Selmi, Manatsaphon Sukmak, Jutarop Phetcharaburanin, Jia V. Li, Atit Silsirivanit, Raynoo Thanan, Amod Sharma, Sirirat Anutrakulchai, Bruce D. Hammock, Ubon Cha’on

**Affiliations:** 1 Department of Biochemistry, Faculty of Medicine, Khon Kaen University, Khon Kaen 40002, Thailand; 2 Chronic Kidney Disease Prevention in the Northeast Thailand (CKDNET), Khon Kaen University, Khon Kaen 40002, Thailand; 3 Rheumatology and Clinical Immunology, IRCCS Humanitas Research Hospital, 20089 Rozzano, Milan, Italy; 4 Department of Biomedical Sciences, Humanitas University, 20072 Pieve Emanuele, Milan, Italy; 5 Department of Metabolism, Digestive Disease and Reproduction, Faculty of Medicine, Imperial College London, South Kensington, London SW7 2AZ, UK; 6 Department of Internal Medicine, Faculty of Medicine, Khon Kaen University, Khon Kaen 40002, Thailand; 7 Department of Entomology and Comprehensive Cancer Research Center, University of California, Davis, CA 95616, USA

We sincerely appreciate the thorough review and insights of Dr. Huichia Chao and colleagues [1] concerning our recent publication [2]. 

First, it is important to clarify that the MSG intake of 1.5 g/kg/day used in our rat models is not equal to 90 g/60 kg BW/day as mentioned in the comment [1] because the animal dose cannot be extrapolated to human equivalent dose (HED) by body weight. In fact, an acceptable conversion dose from animal species to another should be normalized by the body surface area (BSA) [3], and this suggests that the dose used in rats (1.5 g/kg/day) in our study equals to 14.6 g/day in humans (Figure 1). Based on the bound form and free form dietary glutamate intake estimated at 15 g/day [4] and the data on a single oral 10 g MSG in healthy human [5], we are convinced that 14.6 g/day is not an unrealistic dose in humans. As a highly polar material, one would anticipate the first-pass excretion and sequestration of MSG. Scaling based on heart rate is a second commonly used technique to compare doses. This method similarly suggests that the rat dose of MSG used is not an unrealistic as a human comparison.

Second, the work by Insawang et al. is a cross-sectional human study that did not evaluate the gut microbiota, but it remains one of very few epidemiological studies available for daily MSG consumption [6], in which 2 g/day, about a half teaspoon/day, of MSG was classified as a low dose of MSG consumption for Thailand. We agree with Peng et al. that the effect of MSG on the human gut microbiota is limited by individual factors and should be applicable only to the small number of participants consuming MSG at 2 g/day [7] and should not be speculated for higher amounts of daily MSG intake [6]. Moreover, the same authors reported the time dependent decline of phylum Verrucomicrobia over 4 weeks of 2 g/day MSG consumption (Table 2) [7]. While we cannot accurately quantify the impact of MSG on the gut microbiota, we are intrigued by the observation that *Akkermansia muciniphila* within the phylum Verrucomicrobia accounts for 1–5% of the gut microbiota [8] and plays a role in the gut barrier protection in obesity and diabetes [9,10,11], both associated with MSG consumption in our previous works. 

Third, we believe that MSG dietary glutamate is not involved in the biosynthesis of TMA in the colon but may increase serum TMAO, the byproduct of TMA metabolism, as reported in rats that received moderate and high salt intake [12] and in mice receiving a high dose of MSG consumption corresponding to 17 g/day for a 70 kg man [13]. While this evidence should not be overinterpreted, TMAO and TMA adversely impact the heart and kidney health, and the possible role of high-dose prolonged MSG consumption should be further investigated.

Fourth, we were probably unclear in describing the effects of high dose of dietary glutamate on the liver metabolites and pointed to the 1971 work by Prosky and Odell. While no major effects were observed in rat liver metabolites such as glutamate, lactate, malate, and alpha-glycerophosphate, a significant change was demonstrated in aspartate. We believe this supports our hypothesis [14]. On the same ground, changes in liver metabolites after MSG consumption were observed in a classical kinetic experiment using ^14^ C tracing and the data illustrated that the carbon-skeleton of MSG is converted to serum glucose, lactate, aspartate and other amino acids [15]. We cannot rule out the possibility that the observation in the 1971 work by Prosky and Odell of unchanged glutamate, lactate, and malate may be due to the kinetic changes, since ingested glutamate increased in serum and reached the peak at 30 min and started to decline and finally disappeared after 2 h. Although Stegink et al. in 1973 did not address liver tissue metabolites, it is well documented that the hepatic gluconeogenesis is the major source of serum glucose. 

Fifth, the argument that “long-term toxicological data at doses of up to 4% in the diet for up to 2 years show no adverse effects of MSG/glutamate on every organ” based on Owen et al., 1978 also warrants a deeper discussion. In this study rats received MSG mixed with diet in different concentration (1, 2, 4 g%, *w*/*w*) for 104 weeks and the manuscript includes only limited hard data to support the conclusion. On the other hand, Table 2 shows a higher incidence of focal mineralization beneath the epithelium of the renal pelvis in the 4 g% MSG (male, 15/27; female, 29/32) compared to controls (male, 0/8; female, 3/8) at 104 weeks’ timepoint [16]. The same group investigated the effects of MSG on the fitness of dogs based on body weight, behavior, ECG, ophthalmology, hematology, blood chemistry and organ weight by mixing the MSG in their diet (2.5, 5, 10 g%, *w*/*w*) for 2 years [17]. Because limited data and no figures are available (except for the conclusion in the text mentioning no obvious abnormal findings), we can only comment on the data from Table 1 in Owen et al., 1978 [17]. Based on the data at 26 weeks, the 24 h urine volume of MSG-treated animals was relatively higher compared to controls and started to decline over time with exposure, especially in the 10% MSG group (Figure 2), thus suggesting that 10% MSG used for 2 years may indeed have an effect on kidney function. 

In conclusion, we are again thankful for the very insightful comments that allowed stimulated discussion on data extending from the origins of this MSG study and possibly added some helpful insight to the arguments. We remain convinced that, albeit human data are still awaited, low-dose MSG consumption may alter the gut barrier protective bacteria and high dose with long-term MSG consumption should be re-evaluated for the safety, especially when there is a high risk for kidney disease. 

## Figures and Tables

**Figure 1:**
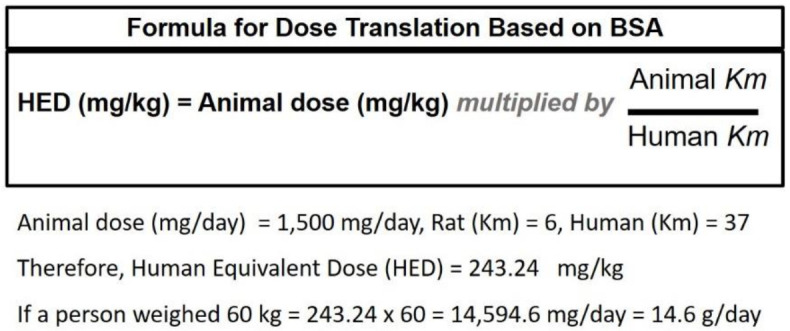
The formula for dose translation based on body surface area (BSA), modified from Reagan-Shaw et al., 2008 [3].

**Figure 2:**
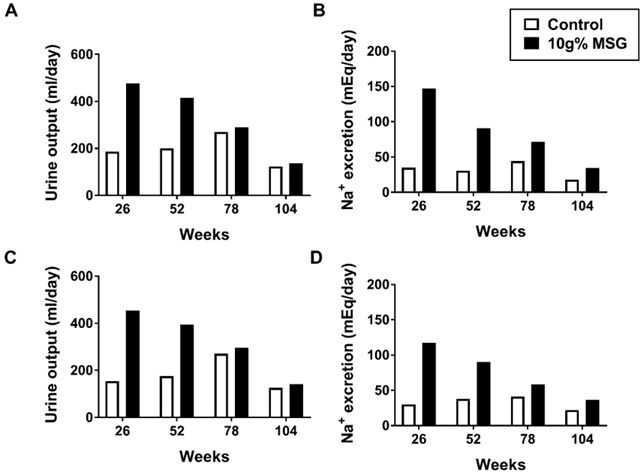
Urine output and sodium excretion in dogs fed with 10 g% MSG for 2 years compared to the controls, modified from Owen et al., 1978 [17]; (**A**) urine output (**B**) sodium excretion in male compared to control groups (*n* = 5, each), (**C**) urine output (**D**) sodium excretion in female compared to control groups (*n* = 5, each).
